# Effects of aqueous extract of turnip leaf (*Brassica rapa*) in alloxan-induced diabetic rats

**Published:** 2015

**Authors:** Mohammad Hassanpour Fard, Ghodratollah Naseh, Nassim Lotfi, Seyed Mahmoud Hosseini, Mehran Hosseini

**Affiliations:** 1 *Department of Physiology and Pharmacology, Birjand University of Medical Sciences (BUMS), Birjand, Iran*; 2 *Department of General Surgery, BUMS, Birjand ,Iran*; 3 *Department of Anatomy, BUMS, Birjand, Iran*; 4 *Department of Biostatistics, BUMS, Birjand, Iran*; 5 *Department of Public Health*, *Research Centre of Experimental Medicine, BUMS, Birjand, Iran*

**Keywords:** *Diabetes mellitus*, *Alloxan*, *Brassica rapa*

## Abstract

**Objectives:**

Turnip leaf has been used in folk medicine of Iran for the treatment of diabetes. However,so far no scientific study has been done to support its use in traditional medicine. The present study was carried out to evaluate the possible hypoglycemic efficacy of aqueous extract of turnip leaf (AETL) in diabetic rats.

**Materials and Methods:**

Alloxan-induced diabetic rats were orally treated with AETL at doses of 200 and 400 mg/kg body weight (bw) per day for 28 days. In order to evaluate the anti-diabetic activity, fasting blood glucose concentrations were determined on the 1^st^, 14^th^ and 29^th^ days. Moreover,at the end of the study, plasma concentrations of total cholesterol, triglyceride (TG), high density lipoprotein cholesterol (HDL-c), low density lipoprotein cholesterol (LDL-c), aspartate amino transfarase (AST), and alanine amino transferase (ALT) were measured by the use of standard kits and auto-analyzer.

**Results:**

Both doses of AETL significantly decreased (*p*<0.001) blood glucose and ALT levels in diabetic rats after 28 days of administration. AETL at both doses decreased (*p*<0.05) plasma total cholesterol and LDL-c in diabetic rats, but they significantly decreased (p<0.05) HDL-c and increased triglycerideand AST levels in a-dose dependent manner.

**Conclusion:**

The results showed that AETL has a dose- dependent decrease in the blood glucose in diabetic rats. However,we should not be unaware of adverse effects of AETL on lipid profiles and liver enzymes activity, especially decrease of HDL and increase of TG and AST.

## Introduction

Diabetes mellitus is a chronic metabolic disease characterized by hyperglycemia due to the impaired secretion and/or action of insulin (Chikhi et al., 2014 ▶).Its prevalence is increasing in many populations all over the world. In 2011, there were 366 million cases with diabetes, and it is expected to increase up to 522 million by 2030(Whiting et al., 2011 ▶). Hyperglycemia leads to alteration in metabolism of carbohydrate, protein, and fat (Wan et al., 2013 ▶). These metabolic disorders induce long term damages and dysfunction of various organs includingeyes, kidneys, nerves, and blood vessels (Santaguida et al., 2005 ▶).There are multiple pharmacological interventions to reduce the hyperglycemia.

Therapy has been based on insulin or drugs that stimulate insulin secretion (sulphonylureas and rapid-acting secretagogues), reducing hepatic glucose production (biguanides), delaying digestion and absorption of intestinal carbohydrate (alpha-glucosidase inhibitors), or improving insulin action in thiazolidinediones(Grossman et al., 2013 ▶).Unfortunately, all of these therapies have various side effects such as gastrointestinal upset, weight changes, hypoglycemia, joint stiffness, kidney complications, and skin alterations(Nathan,2007 ▶; Soccio et al., 2014 ▶).

The prevalence of complementary and alternative medicine use among people with diabetes ranges from 17 to 72.8%. The most widely used therapies among diabetic populations are nutritional supplements, herbal medicine, nutritional advice, spiritual healing, and relaxation techniques. Evidence suggests that a high proportion of people with diabetes use these therapies concurrently with conventional health care services (Chang et al., 2007 ▶).Natural compounds have been proposed for prevention and/or treatment of diabetes. They act viainsulin-like activity, promoting glucose transport, and glucose metabolism (Alberti et al., 2006 ▶; Lee et al., 2006 ▶)

Plants of Brassicacea family play a major role in worldwide vegetable production and consumption. Among them,*Brassica rapa* (turnip) has been cultivated for many centuries across Europe expanding eventually to central and east Asia (Dixon, 2006 ▶). Turnip parts (root, leave, and seed) have been used in traditional medicine commonly for the treatment of some diseases such as diabetes (Javadzadeh and Pouyan, 2010 ▶).

Turnip leaf contains biologically active compounds such as flavonoids including isorhamnetin, kaempferol and quercetin glycosides, phenyl propanoid derivatives, indole alkaloids, and sterol glucosides (Romani et al.,2006 ▶; Schonhof et al., 2007 ▶).Several studies have been reported that polyphenols and flavonoids have beneficial effects particularly on diabetes (Limet al.,2006 ▶). 

However, the impact of turnip leaf in diabetes has not been elucidated. The present study was conducted to evaluate the possible hypoglycemic and hypolipidemic effects of aqueous extract of turnip leaf(AETL) in alloxan-induced diabetic rats.

## Materials and Methods

**Plant collection and extract preparation**

The leaves of *Brassica rapa* were collected during December 2011 from south Khorasan province, Birjand, Iran. The leaves were identified by an expert botanist, and a voucher specimen (221) was kept in the herbarium of agricultural faculty of Birjand University, Birjand, Iran.

*Brassica rapa* leaves were allowed to dry in shade. Dried leaves were powdered by electric grinder (Moulinex AR1043-UK).Powdered leaves weremacerated in distilled water 1:10(w/v) for 2 days at room temperature. Afterwards, the mixture was filtered **(Blue Ribbon, Grade 589,** Germany), from which 10 ml of concentrated extract was transferred and dried in a Petri dish at a temperature of 40 **°**C. The yield of the dried extract was 15.7 g per 100 g of dried turnip leaves. In this study, aqueous extract was used because in folk medicine people consume the infusion of turnip leaf.

**Phytochemical screening**

In order to determine the presence of alkaloids, glycosides, flavones, saponins, and tannins, preliminary phytochemical study of the aqueous extract of turnip leaf was performed (Tiwani et al., 2011 ▶).Polyphenol content was also determined spectrophotometricallyusing Folin-Ciocalteu's method as described by Zivkovic et al. (Zivkovic et al., 2006 ▶). Gallic acid was used as standard to measure the total polyphenol content in the extract.

**Animals and drugs**

In this experimental study, male albino Wistar rats of body weight 180-220 g were obtained from Pastor Institute, Iran. Animals were housed in polyethylene cages at temperature 21-25 **°**C, 12 h light/dark cycle and relative air humidity 40-45%. Rats had continuous access to standard commercial food (JavanehCo, Iran) and tap water. The experimental procedure used in the present work was approved by the Ethic Committee of the animal laboratory of Birjand University of Medical Sciences (BUMS), Birjand, Iran.

Alloxan was obtained from Sigma Co,USA and metformin tablets from Merck Sante' s.a.s., Lyon, France. Alloxan and metformin were freshly dissolved in normal saline solution for intraperitoneal and oral administration, respectively.

**Induction of diabetes and experimental design**

Diabetes was induced by an intraperitoneal injection of freshly prepared alloxan monohydrate dissolved in normal saline at the single dose of 150 mg/kgbody weight (bw) to overnight fasted rats. After 14 days of alloxan administration, the rats with fasting blood sugar (FBS) concentrations more than 350 mg/dl were allocated as severe diabetic (Etuk, 2010 ▶).

40 male Wistar rats were randomly divided into five equal groups (four diabetic and one healthy group). Normal saline solution was administered orally in healthy and diabetic controls rats at the same volume. Metformin at the dose of 50 mg/kg bw was administrated orally in diabetic rats as positive control group. The extract was dissolved in normal saline and daily administered orally in diabetic rats at the doses of 200 and 400 mg/kg bw for 28 days. The selected doses in this study were similar to several other studies (Ezuruike and Prieto, 2014 ▶).

**Estimation and blood samples**

Blood samples were obtained by amputation of the tail tip of 14-hour fasted rats. FBS concentrations were measured on1^st^, 14^th^, and 29^th^ days using a glucometer (AccuChek Active,Germany). On 29^th^ day, 24h after thelast administration, overnight fasted rats were anesthetized and blood samples were drawn from their heart. Total cholesterol(TC), triglyceride (TG), high density lipoprotein cholesterol (HDL-c), low density lipoprotein cholesterol (LDL-c), aspartate amino transferase (AST), and alanine amino transferase (ALT) were estimatedby the use of standard kits (Pars Azmun company, Iran) and auto-analyzer (Prestige 24i- Japan).

**Statistical analysis**

All the data were expressed as mean ± Standard Deviation (SD) except the values of AST and ALT which were shown as mean ± Standard Error of Mean (SEM). Data were analyzed using one-way ANOVA and tukey’spost-hoc test. Values were considered significantly different at p<0.05.

## Results

There was a significant elevation of blood glucose, total cholesterol, triglyceride, LDL-c, AST, and ALT levels in diabetic control rats as compared with non-diabetic control group.

**Effect of AETL on blood glucose concentration in diabetic rats**

Table1shows significant elevation of glucose concentrations on the first dayamong investigation groups that received alloxan. After 14 days of administration, the extract at the dose of 400 mg/kg and positive control group that received metformin 50mg/kg significantly decreased (*p*<0.001) blood glucose concentration compared with diabetic control rats but there was not a significant decrease in group 4 that was treated with 200 mg/kg bw of the extract. On the 29^th^ day, both doses of the extract and metformin significantly decreased (*p*<0.001) blood glucose levels when compared with diabetic control group. There was no significant difference (p>0.05) between groups metformin and the extract at the dose of 400mg/kg on the 29^th^ day.

**Effect of AELT on plasma AST and ALT activities in diabetic rats**

**Figure1:**
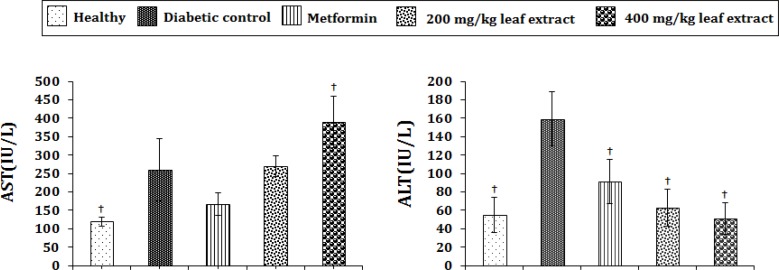
Effect of the aqueous leaf extract of *Brassica rapa *on plasma aspartate amino transferase(AST)and alanine aminotransferase (ALT) activities in diabetic rats.Values are given as mean ± S.E.M, n=8. ^†^ p<0.001 compared to diabetic control group

Figure 1 (A) shows that the administration of AELTat the dose of 200 mg/kg bw did not change AST levels in diabetic rats, while it increased the level of AST at the dose of 400 mg/kg bw. Metformin at the dose of 50 mg/kg bw significantly decreased (*p*<0.05) AST in diabetic rats. 

Figure 1(B) illustrates that the administration of AELT in both doses and metformin significantly decreased (p<0.001) ALT levels compared to diabetic control rats.

**Table 1:** Effect of *Brassica rapa *aqueous leaf extract on blood glucose concentration

	**Blood glucose concentration (mg/dl) Means±SD**		
**Groups (n=8)**	**First day**	**14** ^th^ ** day**	**29** ^th^ ** day**
1-Healthy	8.22†±101.62	5.74†±100.87	4.94†±103.25
2-Diabetic control	461.50 ± 47.07*	466.75 ± 48.73*	515.00 ± 88.80*
3-Diabetic+metformin 50mg/kgbw	471.78 ±29.94*	170.87 ± 46.48†	113.25 ± 14.05†
4-Diabetic+200mg/kg bw leaf extract	47.97*± 466.12	88.96*± 390.00	63.20*†± 312.25
5-Diabetic+400mg/kg bw leaf extract	36.01*± 469.62	127.77*†± 272.87	100.61†±188.62

* p<0.001 compared with normal control group,

† p<0.001 compared with diabetic control group

**Table 2:** Antihyperlipidemic effect of aqueous leaf extract of *Brassia rapa* in diabetic rats.

**Groups (n=8)**	**Cholesterol** **(mg/dl)**	**Triglyceride** **(mg/dl)**	**HDL** **(mg/dl)**	**LDL** **(mg/dl)**
1-Healthy	81.62± 12.29†	73.75± 6.04†	31.50 ± 5.07	30.62 ± 8.12†
2-Diabetic control	112.25±21.68*	114.50 ± 5.39*	33.25 ± 5.77	55.12 ± 18.86*
3-Diabetic+metformin 50mg/kg bw	112.25± 12.09*	85.50 ± 20.88	37.12 ± 3.06	51.87±11.77*
4-Diabetic+200 mg/kg bw leaf extract	62.12± 12.07†	109.25 ± 9.42*	22.78 ± 4.32†	17.75 ± 5.54†
5-Diabetic+400 mg/kg bw leaf extract	65.50±14.51†	127.25 ± 21.96†*	22.50 ± 5.68†*	16.87 ± 6.01†

* p<0.001 compared with normal control group

† p<0.001 compared with diabetic control group

**Effect of AELT on plasma lipid profile in diabetic rats**

Administration of alloxan significantly elevated (*p*<0.05) TC, TG, and LDL-c levels in diabetic rats (Table 2). There was no significant difference in HDL-c between diabetic control group and healthy control group. However, both doses of the extract significantly decreased (p<0.05) total cholesterol and LDL–c levels but increased TG (especially at the dose of 400 mg/kg) and decreased HDL-c at both doses significantly (p<0.05). 

**Phytochemical screening of aqueous extract of turnip leaf**

The qualitative preliminary phytochemical screening showed that the aqueous extract of *Brassica rapa* contained flavonoids and tannins while saponins, glycosides, and alkaloids were absent. The mean of three parallel replicates of total polyphenol content in aqueous extract was 20.38 ± 0.72 mg/g gallic acid equivalent (GAE). 

## Discussion

The present study showed that AELT has the ability to significantly decrease serum glucose and prevent to elevation of plasma ALT in a dose-dependent manner. It also prevented total cholesterol and LDL-c elevation at both doses in comparison to control diabetic rats.

According to our findings, AELTina dose-dependent manner significantly decreased HDL cholesterol and increased AST as well as triglyceride in diabetic rats.

Diabetes can be induced by pharmacological, surgical, or genetic manipulation in several animal species especially in rodents (Bliss,2007 ▶). The majority of studies have usedpharmacological models in which streptozotocin or alloxan most frequently used for induction of diabetes (Shirwaikar et al.,2006 ▶). Both drugs exert their diabetogenic action through reactive oxygen species, which cause rapid destruction of pancreatic β-cells (Szudelski, 2001 ▶). In the present study, alloxanwas used to induce diabetes in animal as previously reported.

Blood glucose in our body is derived from three sources, i.e.,intestinal absorption of dietary carbohydrates, glycogenolysis, and gluconeogenesis (Giugliano et al., 2008 ▶).Due to insulin deficiency (secretion or action) gluconeogenesis rises and subsequentlyliver production of glucose increases (Liu et al., 2010 ▶). It has been suggested that in insulin-dependent diabetes, glucose uptake into skeletal muscle and adipose tissues is impaired (Gonzalez et al., 2006 ▶). Moreover,in experimental diabetes models, intestinal carbohydrate digestion and absorption are altered which cause increasing of glucose uptake from the gut (Goto et al., 2012 ▶).

Anti-hyperglycemic activity of turnip leaf may be due to possession of high levels of polyphenolic compounds and presence of flavonoids and tannins in this extract. Other studies have shown that some of anti-diabetic medicinal plants such as garlic, onion and fenugreek are rich inpolyphenol compounds(El-Demerdash et al., 2005 ▶;Jelodar et al., 2005 ▶).In our study polyphenol amountof AELTwas 20.38 ± 0.72 mg/g GAEwhich iscomparable to garlic and onion (Lu et al., 2011 ▶; Cheng et al.,2013 ▶; Seasotiya et al.,2014 ▶). Another study showed that edible parts of turnip (leave, root, and flower) contain 14 phenolic components and 6 organic acids (Fernandes et al., 2007 ▶). 

AMP-activated protein kinase (AMPK)pathway is an important sensor of cellular energy status and has a key role in the metabolic control.Therefore,it is considered as a new treatment for obesity, diabetes, and metabolic syndrome andis themain target for anti-diabetic drugs including metformin (Kumaret al.,2009 ▶; Zang et al.,2006 ▶).Some polyphenolsimprove glucose uptake in muscle cells and adipocytes by translocation of glucose transporter,GLUT4,to plasma membrane mainly through induction of the AMP-activated protein kinase pathway(Park et al.,2007 ▶; Zhang et al., 2011 ▶).Polyphenols also inhibit α-glucosidase and α-amylase,theenzymes responsible for digestion of dietary carbohydrate to glucose(Taderaet al.,2006 ▶). Plant-food polyphenols have shown to attenuate hepatic gluconeogenesis via decreasing activity of glucose-6-phosphatase and phosphor endol pyruvate carboxykinase (PEPK) causing down-regulation of liver glucokinase (Waltner-Law et al., 2002 ▶). Moreover,some polyphenols protect β-cells from oxidative damages by enhancing the natural antioxidant system and inhibition of lipid peroxidation (Szkudelski,2006 ▶; Szkudelski &Szkudelska.,2001 ▶).It was reported that flavonoids protect normal rat islets from alloxan, normalizes blood glucose levels, and promotes β-cell regeneration in islets of alloxan-treated rats (Vessal et al.,2003 ▶). Exposure of isolated rat islets to certain flavonoids such as(y)-epicatechin or quercetin enhances insulin release by 44–70%(Tabatabaei-Malazy et al., 2013 ▶).

In summary, as per the above-mentioned reasons, hypoglycemic activity of turnip leaf may be due to stimulating of peripheral glucose uptake in tissues, decreasing liver gluconeogenesis, regulating carbohydrate metabolism, and attenuating intestinal absorption of dietary carbohydrate. Therefore, turnip leaf chemical components may have exert regenerative effect on β cellsandstimulate these cells to produce more insulin or have some insulin-like substances.

Enzymes directly associated with the conversion of aminoacids to ketoacids are AST and ALT (Yin et al.,2011 ▶). As a result of damage or toxicity to the liver (*e.g.,* in diabetic patients), these enzymes may leak from hepatocytes into circulationwhichcan lead to elevation in blood(Arobene et al.,2013 ▶).In our study, similar to several other studies, AST and ALT were significantly elevated in diabetic rats in comparison to control ones (Karthikand Ravikumar,2011 ▶; Erejuwa etal.,2012 ▶).Despite the fact that aqueous extract of turnip leaf in adose-dependent manner caused significant reduction in the plasma ALT,AST levels were significantly increased in both doses even more than diabetic control group. 

Hyperglycemia and Hyperlipidemia arethe common characteristics of alloxan-induced diabetes in rats(Asgary et al.,2014 ▶).According to our findings, turnip leaf prevented elevation of total cholesterol and LDL-c in diabetic rats. 3-hydroxy-3-methyl-glutaryl coenzyme A reductase (HMG CoA reductase) is the rate-regulatory enzyme of cholesterol biosynthesis (Sharma et al.,2003 ▶). Lipid lowering effect of the extract might be due to inhibitory effect of flavonoids and polyphenols compounds of turnip leaf on HMG CoA reductase which can lead to the reduction in cholesterol levelor by stimulating effect of glucose utilization in peripheral tissues (Yang et al., 2010 ▶). Normally, lipoprotein lipase is activated by insulin.Therefore, insulin resistance/deficiency may result in extremely elevated triglyceride levels (Basciano et al., 2005 ▶). In the present study, turnip leaf extract in both doses did not inhibit elevation of triglyceride in diabetic rats.

According to our knowledge, this is the first study that evaluated hypoglycemic efficacy of turnip leaf in alloxan-induced diabetic rats. Other studies that investigated the effects of root parts of *Brassica rapa* showed hypoglycemic effect and decreased ALT, AST, and cholesterol inthe treated animals (Jung et al., 2008 ▶; Mohajeri et al, 2011 ▶).

Finally, our results support the folk medicine recommendations to the use of *Brassica rapa* leaves as an anti-diabetic herbal medicine. These findings also provide a warning for its hypoglycemic toxicity potential (ASTandTG increasing andHDL lowering) for the diabetic peoples who consume this plant. Further studies should be carried out to confirm possible actions and mechanisms of the mentioned side effects ofturnip leaf. 

## References

[B1] Alberti KG, Zimmet P, Shaw J (2006). Metabolic syndrome:a new world-wide definition A Consensus Statement from the International Diabetes Federation. Diabet Med.

[B2] Arobene A, Braga F, Roraas T, Sandberg S, Bartlett WA (2013). A systematic review of data on biological variation for alanine aminotransferase, aspartate aminotransferase and gamma-glutamyl transferase. ClinChem Lab Med.

[B3] Asgary S, Rafieian-Kopaei M, Shamsi F, Najafi S, Sahebkar A (2014). Biochemical andhistopathological study of the anti-hyperglycemic and anti-hyperlipidemic effects of cornelian cherry (Cornus mas L) in alloxan-induced diabetic rats. J Complement Integr Med.

[B4] Basciano H, Federico L, Adeli K (2005). Fructose, insulin resistance, and metabolic dyslipidemia. Nutr Metab (Lond).

[B5] Bliss M (2007). The Discovery of Insulin.

[B6] Chang HY, Wallis M, Tiralongo E (2007). Use of complementary and alternative medicine among people living with diabetes: literature review. J AdvNurs.

[B7] Cheng A, Chen X, Jin Q, Wang W, Shi J, Liu Y (2013). Comparison of Phenolic Content and Antioxidant Capacity of Red and Yellow Onions. Czech J Food Sci.

[B8] Chikhi I, Allali H, El Amine Dib M, Medjdoub H, Tabti B (2014). Antidiabetic activity of aqueous leaf extract of Atriplexhalimus L (Chenopodiaceae) in streptozotocin–induced diabetic rats. Asian Pac J Trop Dis.

[B9] Dixon GR (2006). Vegetable Brassicas and related crucifers.

[B10] El-Demerdash FM, Yousef MI, El-Naga NI (2005). Biochemical study on the hypoglycemic effects of onion and garlic in alloxan-induced diabetic rats. Food Chem Toxicol.

[B11] Erejuwa O, Sulaiman S, Wahab M, Sirajudeen K, Salleh M, Gurtu S (2012). Hepatoprotective effect of tualang honey supplementation in streptozotocin-induced diabetic rats. IntJ ApplResNatProd.

[B12] Etuk EU (2010). Animal models for studying diabetes mellitus. AgrBiol JN Am.

[B13] Ezuruike U F, Prieto J (M). 2014. The use of plants in the traditional management of diabetes in Nigeria: Pharmacological and toxicological considerations. J Ethnopharmacol.

[B14] Fernandes F, Valentao P, Sousa C, Pereira JA, Seabra RM, Andrade PB (2007). Chemical and antioxidative assessment of dietary turnip (Brassica rapa var rapa L). Food Chem.

[B15] Giugliano D, Ceriello A, Esposito K (2008). Glucose metabolism and hyperglycemia. Am J ClinNutr.

[B16] Gonzalez E, McGraw TE (2006). Insulin signaling diverges into Akt-dependent and -independent signals to regulate the recruitment/docking and the fusion of GLUT4 vesicles to the plasma membrane. MolBiol Cell.

[B17] Goto T, Horita M, Nagai H, Nagatomo A, Nishida N, Matsuura Y, Nagaoka S (2012). Tiliroside, a glycosidic flavonoid, inhibits carbohydrate digestion and glucose absorption in the gastrointestinal tract. MolNutr Food Res.

[B18] Grossman A, Johannsson G, Quinkler M, Zelissen P (2013). Therapy of endocrine disease: Perspectives on the management of adrenal insufficiency: clinical insights from across Europe. Eur J Endocrinol.

[B19] Jelodar G, Maleki M, Motadayen M, Sirus S (2005). Effect of fenugreek, onion and garlic on blood glucose and histopathology of pancreas of alloxan-induced diabetic rats. Indian J Med Sci.

[B20] Javadzadeh SM, Pouyan M (2010). Medicinal plants in Qaenat.

[B21] Jung UJ, Baek NI, Chung HG, Bang MH, Jeong TS, Lee KT, Kang YJ, Lee MK, Kim HJ, Yeo J, Choi MS (2008). Effect of the ethanol extract of the roots of Brassica rapa on glucose and lipid metabolism in C57BL/KsJ-db/db mice. ClinNutr.

[B22] Karthik D, Ravikumar S (2011). A study on the protective effect of Cynodon dactylon leaves extract in diabetic rats. Biomed Environ Sci.

[B23] Kumar R, Balaji S, Uma TS, Sehgal PK (2009). Fruit extracts of Momordica charantia potentiate glucose uptake and up-regulate Glut-4, PPAR gamma and PI3K. J Ethnopharmacol.

[B24] Lee YS, Kim WS, Kim KH, Yoon MJ, Cho HJ, Shen Y, Ye JM, Lee CH, Oh WK, Kim CT, Hohnen-Behrens C, Gosby A, Kraegen EW, James DE, Kim JB (2006). Berberine, a natural plant product, activates AMP-activated protein kinase with beneficial metabolic effects in diabetic and insulin-resistant states. Diabetes.

[B25] Lim SS, Jung YJ, Hyun SK, Lee YS, Choi JS (2006). Rat lens aldose reductase inhibitory constituents of Nelumbonucifera stamens. Phytother Res.

[B26] Liu SH, Chang YH, Chiang MT (2010). Chitosan reduces gluconeogenesis and increases glucose uptake in skeletal muscle in streptozotocin-induced diabetic rats. J Agric Food Chem.

[B27] Lu X, Ross CF, Powers JR, Aston DE, Rasco BA (2011). Determination of Total Phenolic Content and Antioxidant Activity of Garlic (Allium sativum) and Elephant Garlic (Allium ampeloprasum) by Attenuated Total Reflectance–Fourier Transformed Infrared Spectroscopy. J Agric Food Chem.

[B28] Mohajeri D, AmouoghliTabrizi B, Doustar Y, Nazeri M, Brassica rapa L (2011). root extract alleviate early hepatic injury in alloxan-induced diabetic rats. J Med plants Res.

[B29] Nathan DM (2007). Finding new treatments for diabetes - How many, how fast how good?. N Engl J Med.

[B30] Park CE, Kim MJ, Lee JH, Min BI, Bae H, Choe W, Kim SS, Ha J (2007). Resveratrol stimulates glucose transport in C2C12 myotubes by activating AMP-activated protein kinase. ExpMol Med.

[B31] Romani A, Vignolini P, Isolani L, Leri F, Heimler D (2006). HPLC-DAD/MS characterization of flavonoids and hydrocinnamic derivatives in turnip top (Brassica rapa L Subsp Sylvestris L). J Agric food Chem.

[B32] Santaguida PL, Balion C, Hunt D, Morrison K, Gerstein H, Raina P, Booker L, Yazdi H (2005). Diagnosis, prognosis, and treatment of impaired glucose tolerance and impaired fasting glucose. Evid Rep Technol Assess (Summ).

[B33] Schonhof I, Krumbein A, Bruckner B (2004). Genotypic effects on glucosinolates and sensory properties of broccoli and cauliflower. Nahrung.

[B34] Seasotiya L, Siwach P, Bai S, Malik A, Bharti P, Dalal S (2014). Free radical scavenging activity, phenolic contents and phytochemical analysis of seeds of Trigonella foenum graecum. Asian Pac J Health Sci.

[B35] Sharma SB, Nasir A, Prabhu KM, Murthy PS, Dev G (2003). Hypoglycaemic and hypolipidemic effect of ethanolic extract of seeds of Eugenia jambolana in alloxan-induced diabetic rabbits. J Ethnopharmacol.

[B36] Shirwaikar A, Rajendran K, Barik R (2006). Effect of aqueous bark extract of GarugapinnataRoxb instreptozotocin-nicotinamide induced type-II diabetes mellitus. J Ethnopharmacol.

[B37] Soccio Raymond E, Chen Eric R, Lazar Mitchell A (2014). Thiazolidinediones and the Promise of Insulin Sensitization in Type 2 Diabetes. Cell Metabolism.

[B38] Szudelski T (2001). The mechanism of alloxan and streptozotocin action in β-cells of the rat pancreas. Physiol Res.

[B39] Szkudelski T (2006). Resveratrol inhibits insulin secretion from rat pancreatic islets. Eur J Pharmacol.

[B40] Szkudelski T, Szkudelska K (2011). Anti-diabetic effects of resveratrol. Ann NY AcadSci.

[B41] Tabatabaei-Malazy O, Larijani B, Abdollahi M (2013). A novel management of diabetes by means of strong antioxidants’ combination. Journal of Medical Hypotheses and Ideas.

[B42] Tadera K, Minami Y, Takamatsu K, Matsuoka T (2006). Inhibition of alpha-glucosidase and alpha-amylase by flavonoids. J Nutr Sci Vitaminol.

[B43] Tiwani P, Kumar B, Kaur G, Kaur H (2011). Phytochrmical screening and extraction: A review. IPS.

[B44] Vessal M, Hemmati M, Vasei M (2003). Antidiabetic effects of quercetin in streptozocin-induced diabetic rats. Comp Biochem Physiol C Toxicol Pharmacol.

[B45] Waltner-Law ME, Wang XL, Law BK, Hall RK, Nawano M, Granner DK (2002). Epigallocatechin gallate, a constituent of green tea, represses hepatic glucose production. J Biol Chem.

[B46] Wan L-s, Chen C-p, Xiao Z-q, Wang Y-l, Min Q-x, Yue Y, Chen J (2013). In vitro and in vivo anti-diabetic activity of Swertia kouitchensis extract. J Ethnopharmacol.

[B47] Whiting DR, Guariguata L, Weil C, Shaw J (2011). IDF Diabetes Atlas: Global estimates of the prevalence of diabetes for 2011 and 2030. Diabetes Res Clin Pract.

[B48] Yang MY, Peng CH, Chan KC, Yang YS, Huang CN, Wang CJ (2010). The hypolipidemic effect of Hibiscus sabdariffa polyphenols via inhibiting lipogenesis and promoting hepatic lipid clearance.

[B49] Yin P, Zhao S, Chen S, Liu J, Shi L, Wang X, Liu Y, Ma C (2011). Hypoglycemic and hypolipidemic effects of polyphenols from burs of Castanea mollissima Blume. Molecules.

[B50] Zivkovic J, Mujic I, Zekovich Z, Nikolic G, Vidovic S, Muji (2009). Extraction and analysis of condensed tannins in Castanea Sativa. J Cent Eur Agric.

[B51] Zang M, Xu S, Maitland-Toolan KA, Zuccollo A, Hou X, Jiang B, Wierzbicki M, Verbeuren TJ, Cohen RA (2006). Polyphenols stimulate AMP-activated protein kinase, lower lipids, and inhibit accelerated atherosclerosis in diabetic LDL receptor-deficient mice. Diabetes.

[B52] Zhang B, Kang M, Xie Q, Xu B, Sun C, Chen K, Wu Y (2011). Anthocyanins from Chinese bayberry extract protect beta cells from oxidative stress-mediated injury via HO-1 upregulation. J Agric Food Chem.

